# Non-destructive prediction and visualization of anthocyanin content in mulberry fruits using hyperspectral imaging

**DOI:** 10.3389/fpls.2023.1137198

**Published:** 2023-03-27

**Authors:** Xunlan Li, Zhaoxin Wei, Fangfang Peng, Jianfei Liu, Guohui Han

**Affiliations:** Research Institute of Pomology, Chongqing Academy of Agricultural Sciences, Chongqing, China

**Keywords:** hyperspectral imaging, mulberry fruit, anthocyanin content, SAE, ELM

## Abstract

Being rich in anthocyanin is one of the most important physiological traits of mulberry fruits. Efficient and non-destructive detection of anthocyanin content and distribution in fruits is important for the breeding, cultivation, harvesting and selling of them. This study aims at building a fast, non-destructive, and high-precision method for detecting and visualizing anthocyanin content of mulberry fruit by using hyperspectral imaging. Visible near-infrared hyperspectral images of the fruits of two varieties at three maturity stages are collected. Successive projections algorithm (SPA), competitive adaptive reweighted sampling (CARS) and stacked auto-encoder (SAE) are used to reduce the dimension of high-dimensional hyperspectral data. The least squares-support vector machine and extreme learning machine (ELM) are used to build models for predicting the anthocyanin content of mulberry fruit. And genetic algorithm (GA) is used to optimize the major parameters of models. The results show that the higher the anthocyanin content is, the lower the spectral reflectance is. 15, 7 and 13 characteristic variables are extracted by applying CARS, SPA and SAE respectively. The model based on SAE-GA-ELM achieved the best performance with R^2^ of 0.97 and the RMSE of 0.22 mg/g in both the training set and testing set, and it is applied to retrieve the distribution of anthocyanin content in mulberry fruits. By applying SAE-GA-ELM model to each pixel of the mulberry fruit images, distribution maps are created to visualize the changes in anthocyanin content of mulberry fruits at three maturity stages. The overall results indicate that hyperspectral imaging, in combination with SAE-GA-ELM, can help achieve rapid, non-destructive and high-precision detection and visualization of anthocyanin content in mulberry fruits.

## 1 Introduction

Mulberry (*Morus* L.) is widely planted around the world. Tender, juicy and delicious mulberry fruits have long been used as traditional medicine as well as edible fruits in countries such as China, India and Turkey (Jan et al., 2021). Modern researches show that black and red mulberry fruits are rich in anthocyanins, which, with the properties of antioxidant, anti-inflammatory and chemical protection, play a positive role in reducing the risk of cardiovascular diseases and cancers (Chen et al., 2006; Krishna et al., 2018). Anthocyanins are considered to be one of the most important indicators for mulberry fruits of good quality by researchers and consumers.

Anthocyanin contents are usually determined by adopting wet chemical methods, such as spectrophotometry (Jiang and Nie, 2015) and high-performance liquid chromatography (Zou et al., 2012). The samples need to be ground and extracted with the use of chemical reagents such as ethanol or acetone. These methods are destructive and will produce chemical residues. And only a small number of samples can be analyzed at a time. It is difficult to detect anthocyanin content in mulberry fruits on a large scale by applying the existing time-consuming and inefficient detecting methods. For efficient agricultural management and production, it is necessary to find a reliable, fast and non-destructive method for anthocyanin content detection.

Hyperspectral imaging (HSI) can obtain the spectral data of each pixel in the sample image simultaneously. This is of potential value in non-destructive detection of uneven distribution of quality indicators. There are reports about visualizing anthocyanin contents of purple sweet potato (Liu et al., 2017), lychee pericarp (Yang et al., 2015), and grape (Chen et al., 2015) by using HSI. The research by Huang et al. (2017) has shown that 400-1000nm and 900-1700nm HSI, in combination with least squares support vector machine (LS-SVM), has great potential in evaluating total anthocyanin content and antioxidant activity of mulberry fruits. This is the only study on determining anthocyanin of mulberry by using HSI. And further research endeavors to visualize anthocyanin content of mulberry fruit have not been reported yet.

The variable selection is an essential step for modeling. From previous researches, variable selection methods, such as interval partial least square, successive projections algorithm (SPA) and competitive adaptive reweighted sampling (CARS) are often used to reduce the number of input variables before modeling (Zhu et al., 2017; Silva and Melo-Pinto, 2021). When using these variable selection methods, the average spectrum of all pixels in the hyperspectral image is applied, while efficient big data analysis of each pixel spectrum is ignored. Depth feature extraction and dimension reduction can be conducted by using the stacked auto-encoder (SAE), a nonlinear unsupervised neural network, which is capable of effectively analyzing the spectral data of all pixels of the hyperspectral image and then select variables (Yu et al., 2018). In terms of modeling, the LS-SVM has been shown to be of good potential in non-destructive detection. Research reports show that the extreme learning machine (ELM), a single hidden layer feedforward neural network model, is able to achieve similar or much better performance at a much faster learning speed than traditional LS-SVM (Huang et al., 2011; Zheng et al., 2014).

This study is meant for developing a rapid, non-destructive, high-precision method to detect and visualize the anthocyanin content of mulberry fruit. The main research objects are as follows: (1) analyzing the differences in anthocyanin content and corresponding spectral data between two mulberry varieties at different maturity stages; (2) reducing the dimension of high-dimensional spectral data by using SPA, CARS and SAE, and selecting the most effective feature variables; (3) using LS-SVM and ELM to build the models for predicting mulberry anthocyanin and selecting the best prediction model so as to achieve rapid, non-destructive and high-precision prediction of the anthocyanin content of mulberry fruit; (4) mapping distribution of anthocyanin content in mulberry fruit.

## 2 Materials and methods

### 2.1 Materials

**Figure 1:**
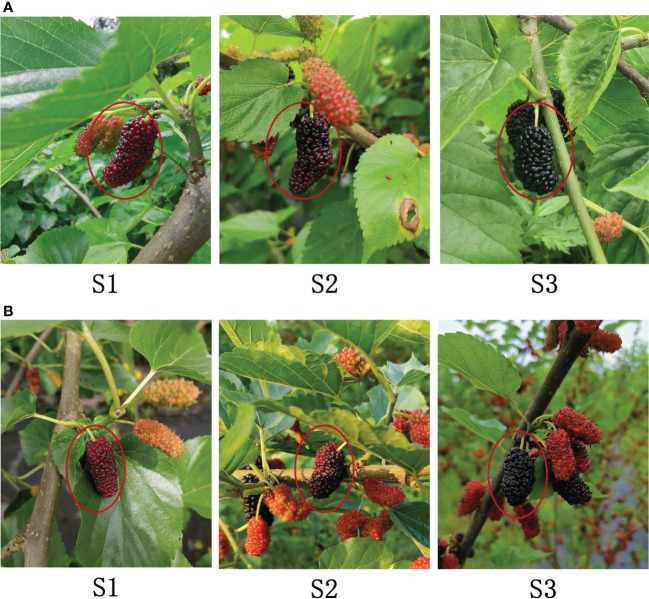
Fruit images of Dashi **(A)** and Siji **(B)** at three maturity stages: (S1) red maturity; (S2) red to purple maturity; (S3) full maturity.

The sampled varieties, Dashi (*Morus nigra* L.) and Siji (*Morus nigra* L.) were collected from the mulberry resource conservation nursery of Chongqing Academy of Agricultural Sciences on April 23, 2020. Disease-free fruits at three maturity stages (S1: red maturity, S2: red to purple maturity and S3: full maturity) were randomly picked, then stored in ice boxes. They were brought back to the laboratory for hyperspectral image collection and anthocyanin content detection (**Figure 1**). Six fruits at the same maturity stage were randomly selected as one sample for anthocyanin content detection. A total of 180 samples were obtained, and the numbers of Dashi and Siji were 90 respectively. The samples were randomly divided into the training set and the testing set at a ratio of 7:3.

### 2.2 Collection and calibration hyperspectral images

**Figure 2:**
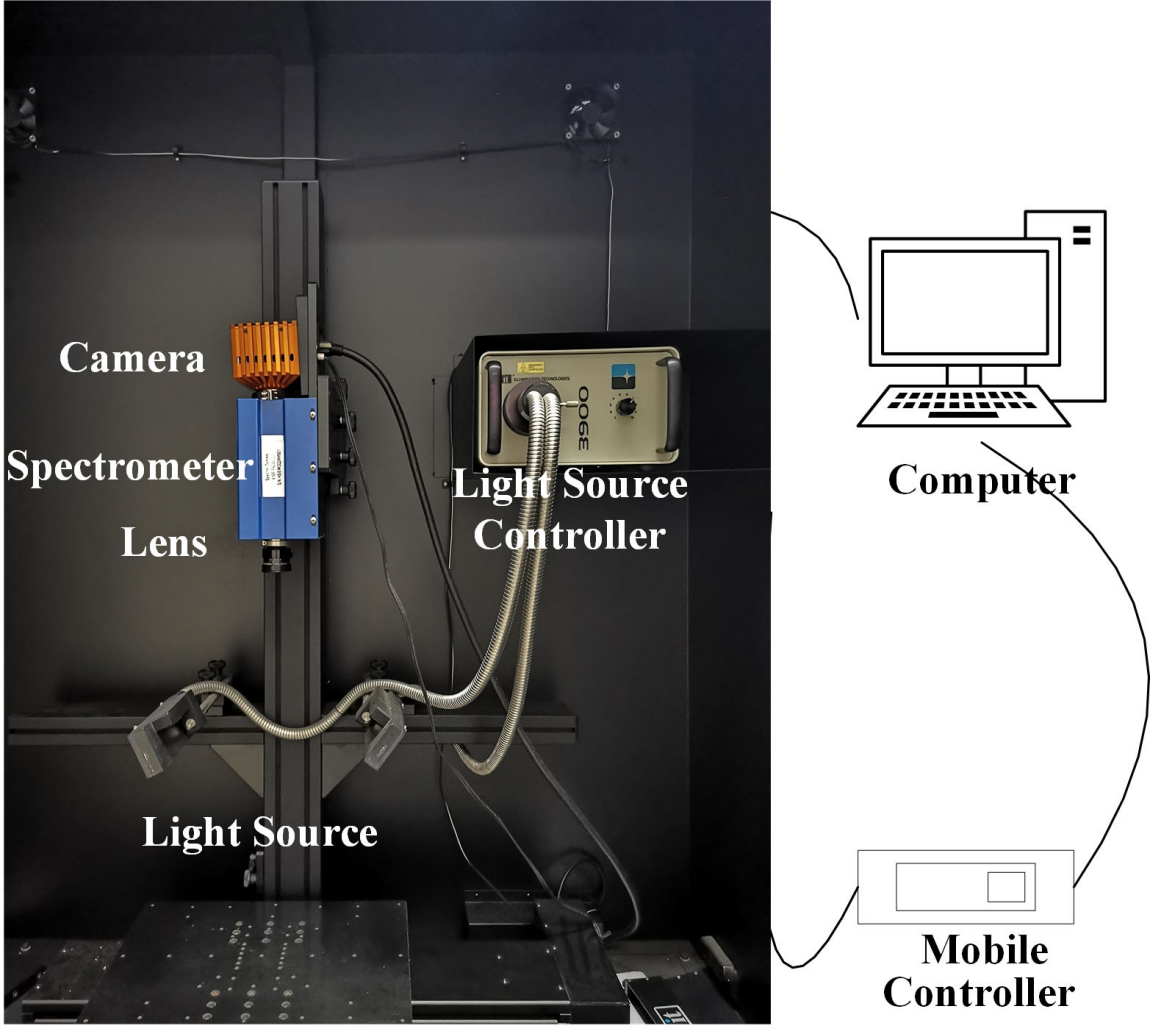
The hyperspectral imaging system.

The hyperspectral imaging system was used to collect hyperspectral images of mulberry fruits (**Figure 2**). The hyperspectral imaging system consists of a spectrograph (ImSpector V10E, SPECIM, Finland), an EMCCD camera (DL-604E, Andor Technology plc., N. Ireland), two halogen light sources (150 W/21 V halogen lamp, Illuminator Technologies, Inc, USA), an electric mobile platform and controller (SC30021A, Zolix, China), and a laptop. The wavelength range of the spectrum collected was 305-1 090 nm. The two light sources were at an angle of 45° with the mobile platform respectively. The camera exposure time was 60 ms. The spectral resolution was 2.8 nm. The platform moving speed was 1.87 mm/s. The distance between the objective lens and the platform was 40 cm. After preheating for half an hour by the light source, the mulberry fruits were placed on the black cardboard for hyperspectral image collection.

The collected hyperspectral images need to be calibrated so as to avoid the effect caused by uneven light source intensity distribution and dark current during the image collecting process. Under the same conditions as the sample images were collected, the white reference image W was obtained by scanning the standard white reference panels. The dark reference image D was obtained by scanning with the lens covered. And the image calibration was completed on the basis of formula (1),


(1) $$R_{\lambda }=\frac{I_{\lambda }-D_{\lambda }}{W_{\lambda }-D_{\lambda }}$$

where *R_λ_* is the calibrated image, *I_λ_* is the raw image, *W_λ_* is the white reference image, and *D_λ_* is the dark reference image.

### 2.3 Anthocyanin content extraction

Anthocyanin content was detected by pH-differential spectrophotometry (Lee et al., 2005). 0.5 ± 0.001 g of grinded fresh mulberry fruits was added to 10 ml of acidified ethanol (95% ethanol and 1% concentrated hydrochloric acid, the volume ratio of ethanol to hydrochloric acid was 60:40) for 1 h ultrasound extraction and 2 min centrifugation at 8000 r•min^-1^. 1 ml of supernatant was taken and the volume was fixed to 25 ml by adding buffer solutions of pH 1.0 and pH 4.5 respectively. The absorbance was measured at 520 nm and 700 nm after letting it stand for 15 min with an ultra-violet-visible spectrophotometer (UV-6000PC ShanhaiMetash. Co. Ltd, China). The anthocyanin content was calculated by the formula (2) and (3).


(2) $$A=(A_{520nm}-A_{700nm})\;at\;PH\;1.0-(A_{520nm}-A_{700nm})\;at\;PH\;4.5$$

(3) Total anthocyanin content(mg/g)=(A*MW*DF*V)/(ϵ*1*M)

Where is the absorbance, *A_520nm_* and *A_700nm_* are the absorbance at the 520 nm and 700 nm respectively. *MW* (molecular weight) = 449.2 g/mol for cyanidin-3-glucoside (cyd-3-glu). *DF* (Dilution factor) = 25. V is the original volume of 10 ml. The molar extinction coefficient *ϵ*=26900. M is the weight of the sample.

### 2.4 Region of interest and spectral data extraction

In this study, the whole fruit with the fruit stalk removed was treated as the region of interest (ROI). The whole mulberry fruit and the collection background plate were segmented at 800 nm, with the reflectance of 0.2 as the minimum value. The petiole was removed from the whole fruit at 550 nm and 670 nm, with the difference value of 0.04 as the maximum value. Then the ROI was obtained by conducting mask processing. The average spectrum of ROI at each wavelength was calculated for subsequent SPA and CARS feature wavelength extraction. To create a data set for deep learning, 400 pixels (20 * 20) corresponding to spectral data were randomly selected from the ROI of each sample, totaling 72,000, for SAE training.

### 2.5 Spectral data processing

#### 2.5.1 Spectral data pretreatment

Owing to the existence of strong noises in the beginning and ending bands of the raw spectral data, spectral data within the range of 450-1050 nm, a total of 379 variables were selected for subsequent analysis. In this study, standard normal variate transform (SNV) was used to preprocess the spectral data, to eliminate the scattering caused by uneven particle distribution and different particle sizes, and the influence of optical path change on the spectral data.

#### 2.5.2 Feature extraction

Successive projections algorithm(SPA), Competitive adaptive reweighted sampling(CARS) and Stacked auto-encoder (SAE) were respectively used in this study to extract spectral data features for the purpose of reducing the number of input variables, improving model efficiency, eliminating redundant information of spectral data, and improving the prediction accuracy of the model.

Successive projections algorithm (SPA) is a forward variable selection algorithm. By this method, the cycle of forward is conducted with a wavelength initially selected and the projection value of the remaining wavelength calculated. Then the projection vector is combined with the wavelength corresponding to the maximum projection value until the cycle ends. The minimum variable group can be effectively obtained by calculating the band projection value, thus minimizing the collinearity between variables (Araújo et al., 2001).

Competitive adaptive reweighted sampling (CARS) is a method based on Monte Carlo sampling and the PLS regression coefficient. By this method, characteristic variables are primarily screened out by using the PLS regression coefficient in combination with the exponential decline function. Then the initially selected characteristic variables are competitively screened out by using adaptive reweighted sampling. And the final characteristic variables are screened out from the wavelength combinations according to the cross-validation root mean square error. The detailed algorithm of CARS can be found in reference (Li et al., 2009). In this study, the number of CARS samples was set to 50, and the ten-fold cross-validation method was used.

Stacked auto-encoder (SAE) is a deep neural network consisting of multilayer auto-encoders (AE), by which better feature extraction is obtained with the hidden layers added to the simple auto-encoders. AE consists of encoders and decoders. The input layers map the input data to the hidden layers through the activation function to obtain the encoding features, which is called encoding. Through the same steps, the encoding features are mapped to the output layers by using the activation function to obtain the decoding features, which is called decoding. In terms of SAE, the decoding features of the previous AE are used as the input of the next hidden layer of AE, and code and decode the next layer of AE. By analogy, these hidden input layers are connected to form SAE (Xu et al., 2022).

#### 2.5.3 Model construction and evaluation

Least squares support vector machine (LS-SVM) is a machine learning algorithm based on SVM, boasting good generalization ability and nonlinear regression processing ability (Suykens and Vandewalle, 1999). The fitting ability of LS-SVM mainly depends on the selection of kernel parameters (C and γ). Kernel parameter C affects the fitting accuracy and generalization ability of the model, and kernel parameter γ directly determines the calculation amount and efficiency of the model.

Extreme learning machine (ELM) is a feedforward neural network with a single hidden layer, which has a fast learning ability and strong nonlinear approximation ability (Huang et al., 2006). Compared with traditional neural network learning algorithms, such as back propagation neural network, ELM presents the advantages of strong generalization ability and fast calculation speed (Ye et al., 2022). Over-fitting is liable to occur, since the weight and offset of ELM are randomly determined.

Genetic algorithm (GA) is a search algorithm for obtaining the global optimal solution based on the biological evolution mechanism of “survival of the fittest” (Mirjalili, 2019). In this study, GA is used to optimize the important parameters of the RBF kernel function and the offset and weight of ELM. In this case, the value ranges of kernel parameters (C and γ) were set to 0.01-100, the population size was set to 20, and the number of maximum evolution times was set to 200. When GA was used to optimize ELM, the population size was set to 20, the maximum number of evolutions was set to 300, and the number of neurons in the hidden layer of ELM was set to 90.

The training set determination coefficient (R^2^c), testing set determination coefficient (R^2^p), training set root mean square error (RMSEC), and testing set root mean square error (RMSEP) were used as indicators to evaluate the performance of models. The closer to 1 the determination coefficient (R^2^) is, the better the model fitting effect is. And the smaller RMSEC and RMSEP are, the higher the precision of the model is.

**Figure 3:**
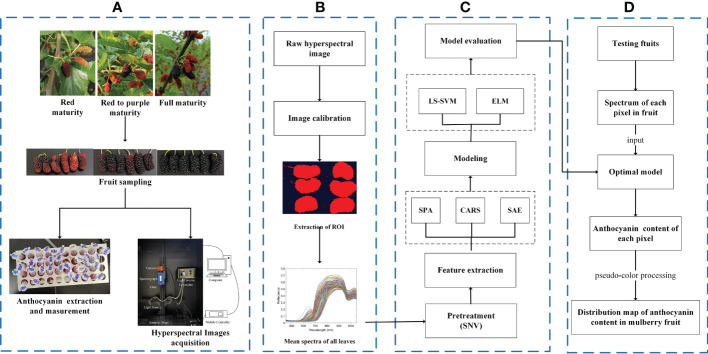
Overall flow chart. **(A)** Acquisition of data; **(B)** hyperspectral image processing; **(C)** analysis of spectral data; **(D)** visualization of anthocyanin content.

The hyperspectral image calibration in this study was completed by the software of the hyperspectral image acquisition system. ROI segmentation, spectral data extraction and processing were completed by using MATLAB 2022a, with SPA, CARS, GA, SAE, and LS-SVM realized by using SPA_GUI, Lib PLS1.98, GATBX, Deep Learning toolbox, and LS-SVMlab v1.8 toolbox. The overall flow is shown in **Figure 3**.

## 3 Results and analysis

### 3.1 Anthocyanin content and spectral characteristics of mulberry fruits

**Figure 4:**
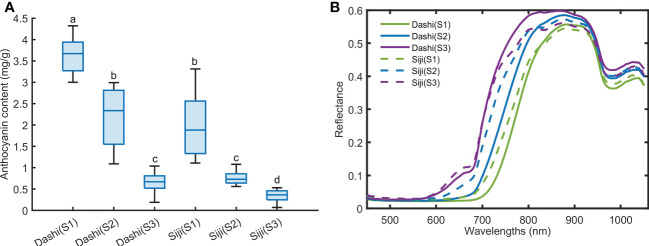
The anthocyanin content **(A)** and average spectra **(B)** of mulberry fruit at three maturity stages. Values with the same letter (i.e. a, b, c or d) are not significantly different (*p*<0.05).

The anthocyanin contents of two mulberry varieties at three maturity stages were analyzed and measured, and the mean anthocyanin content and corresponding spectral reflectance of two mulberry varieties at different maturity stages were calculated (**Figure 4**). It was shown in **Figure 4A** that the higher the maturity of mulberry fruits was, the higher the anthocyanin content was, which followed the description of the report of Saracoglu (Saracoglu, 2018). The anthocyanin content of Dashi was higher than that of Siji at the same maturity stage. Anthocyanins are the main reasons why mulberry has red and purple (Li et al., 2020). From the analysis of the spectrum reflection curve of mulberry fruit, it can be seen that the spectral reflection value in the range from 500 to 700 nm was very low. According to qin and Lu (Qin and Lu, 2008), the maximum absorbance of anthocyanin pigments is about 535 nm. However, the difference between the mulberry fruits of different maturity was not obvious at 535 nm in **Figure 4B**. This may be because the black substances have strong absorption in the visible light area, and the reflectance value is not attributed to a single compound, the spectra are the sum of the major mulberry fruit composition spectra (Cozzolino et al., 2004). A small reflective valley could be seen near 680 nm in red maturity fruits, which is related to the existence of chlorophyll. The spectral reflectance was lower with the increase of maturity and anthocyanin content within the range of 590-800 nm. The two varieties showed obvious absorption peaks near 970 nm and 840 nm. This is speculated to be related to water and sugar absorption (ElMasry et al., 2008; Zheng et al., 2008). The differences in spectral characteristics of the mulberry fruits above show that hyperspectral imaging has the potential to distinguish the mulberry fruits of different anthocyanin contents.

### 3.2 The results of feature extraction

When hyperspectral imaging is used to detect the anthocyanin contents of mulberry fruits, the redundant information is often eliminated and the amount of calculation is compressed by screening out the characteristic wavelengths to improve the accuracy and robustness of the diagnostic models. In this paper, SPA, CARS and SAE were used to extract feature variables from the 379 variables.

**Figure 5:**
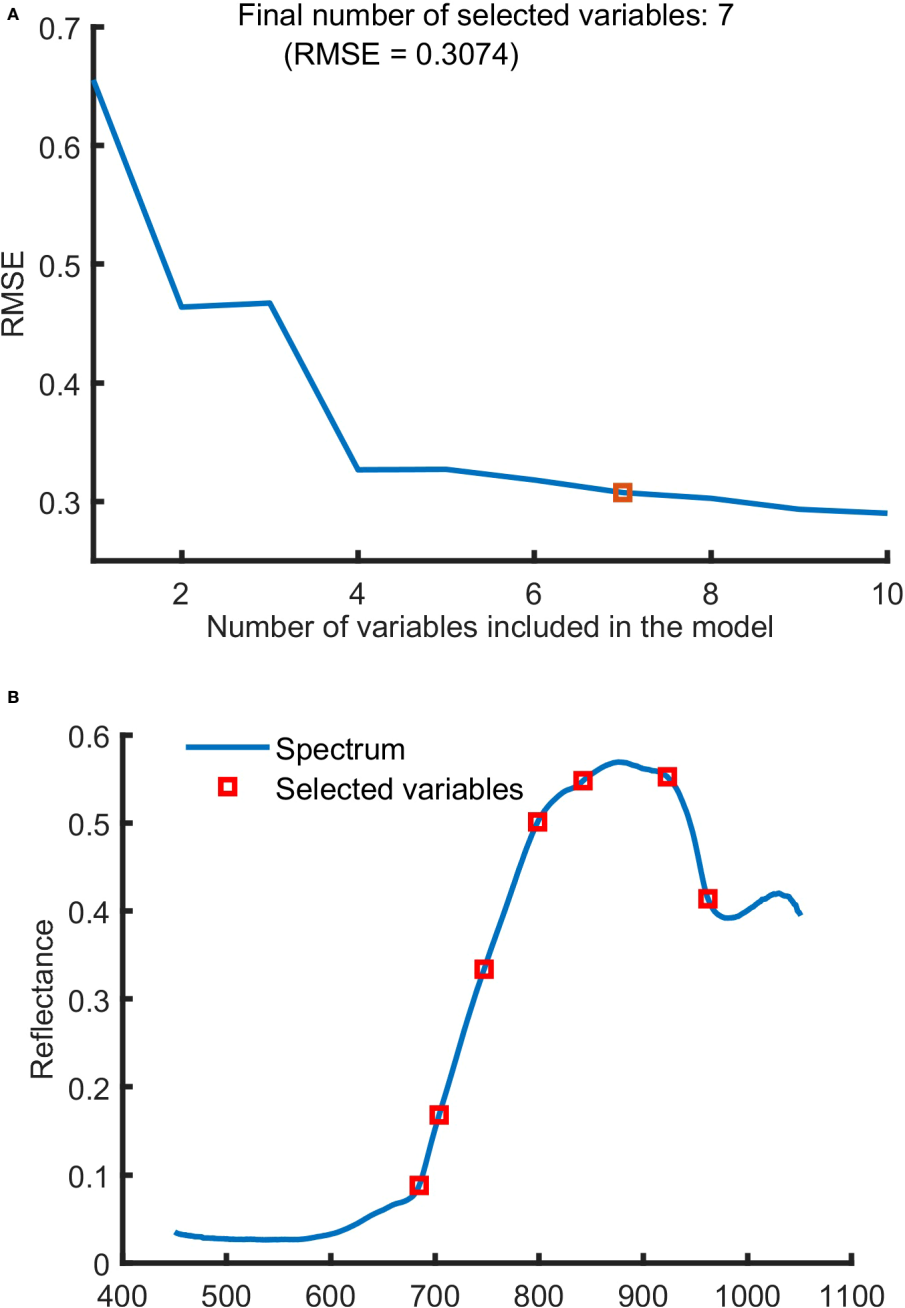
The characteristic wavelengths selected by SPA. **(A)** Variation of RMSE with the number of variables, **(B)** the selected wavelengths.

SPA was used to screen characteristic wavelengths from spectral data of SNV pretreatment in 450-1,050 nm region, and the results were shown in **Figure 5**. It can be seen from **Figure 5A** that when the number of characteristic wavelengths increased from 1 to 7, the value of RMSE decreased in a ladder shape and then leveled off. And 7 characteristic wavelengths at 684.88, 703.98, 747.15, 798.58, 842.15, 923.11 and 962.05 nm were obtained.

**Figure 6:**
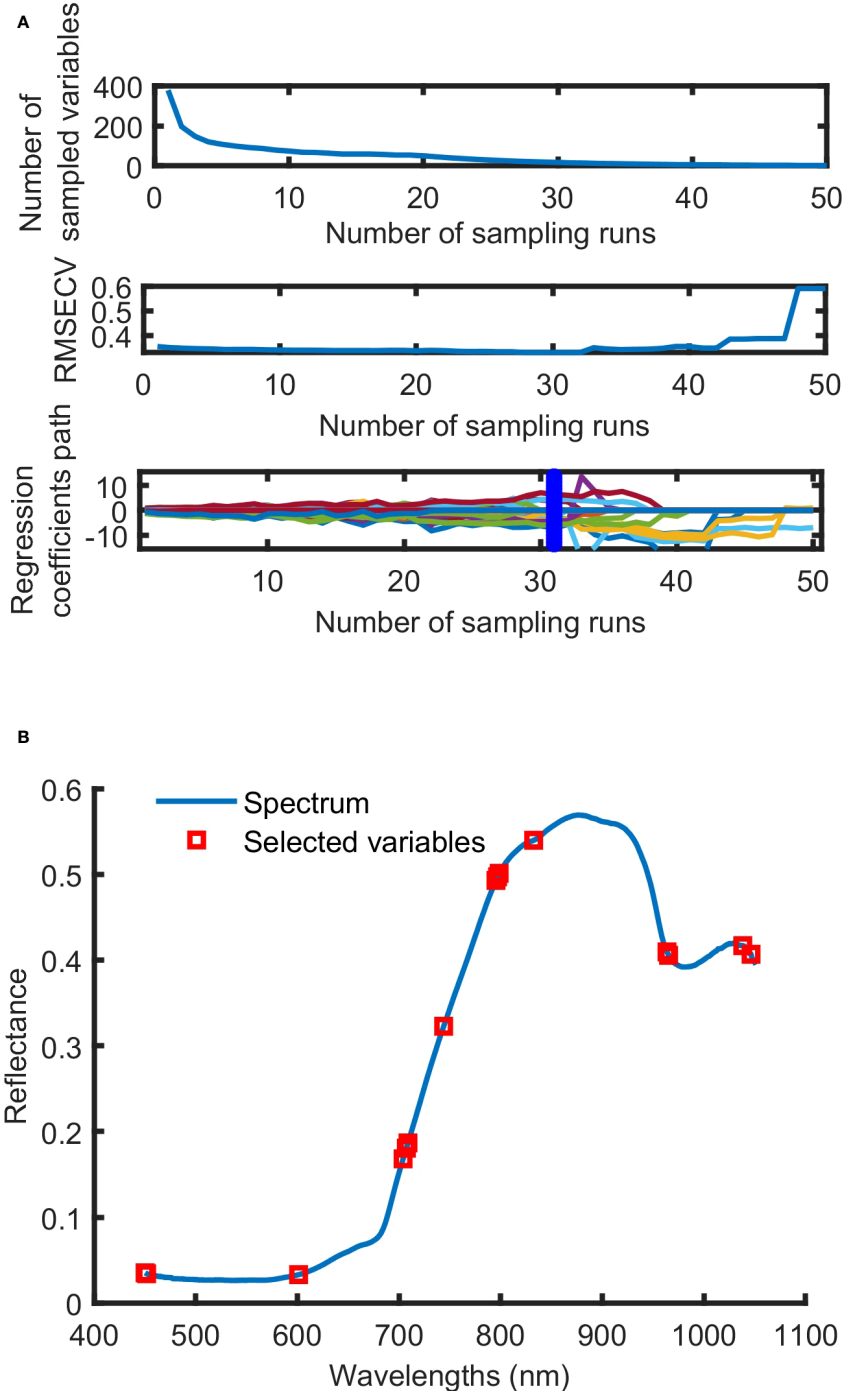
The process **(A)** and result **(B)** of characteristic wavelength selection by CARS.

The process of screening wavelengths by using CARS was shown in **Figure 6**. With the increase in sampling times, the number of selected wavelengths decreased gradually at the speed from fast to slow. This reflected the two stages, preliminary screening and fine screening, of using CARS to screen out key variables. With the increase in sampling times, the root mean squares error of cross-validation (RMSECV) value gradually decreased. And the RMSECV value obtained was the lowest when the 31st sampling was conducted. This is an indication that some irrelevant variables are removed during the sampling process. After the 31st sampling, the RMSECV value presented a stepwise progression, indicating the removal of some key information. Therefore, the wavelengths obtained at the 31st sampling were the characteristic wavelengths. Fifteen characteristic wavelengths, 450.08, 451.59, 601.16, 703.98, 707.17, 708.77, 743.95, 795.36, 796.97, 798.58, 832.45, 963.67, 965.29, 1038.29 and 1046.39 nm, were screened out by using CARS.

Based on the analysis of the characteristic wavelengths, the positions and numbers of characteristic wavelengths screened out by using SPA and CARS were found to be different. And the wavelength positions are concentrated within the ranges of 703-835 nm and 963-1046 nm.

**Figure 7:**
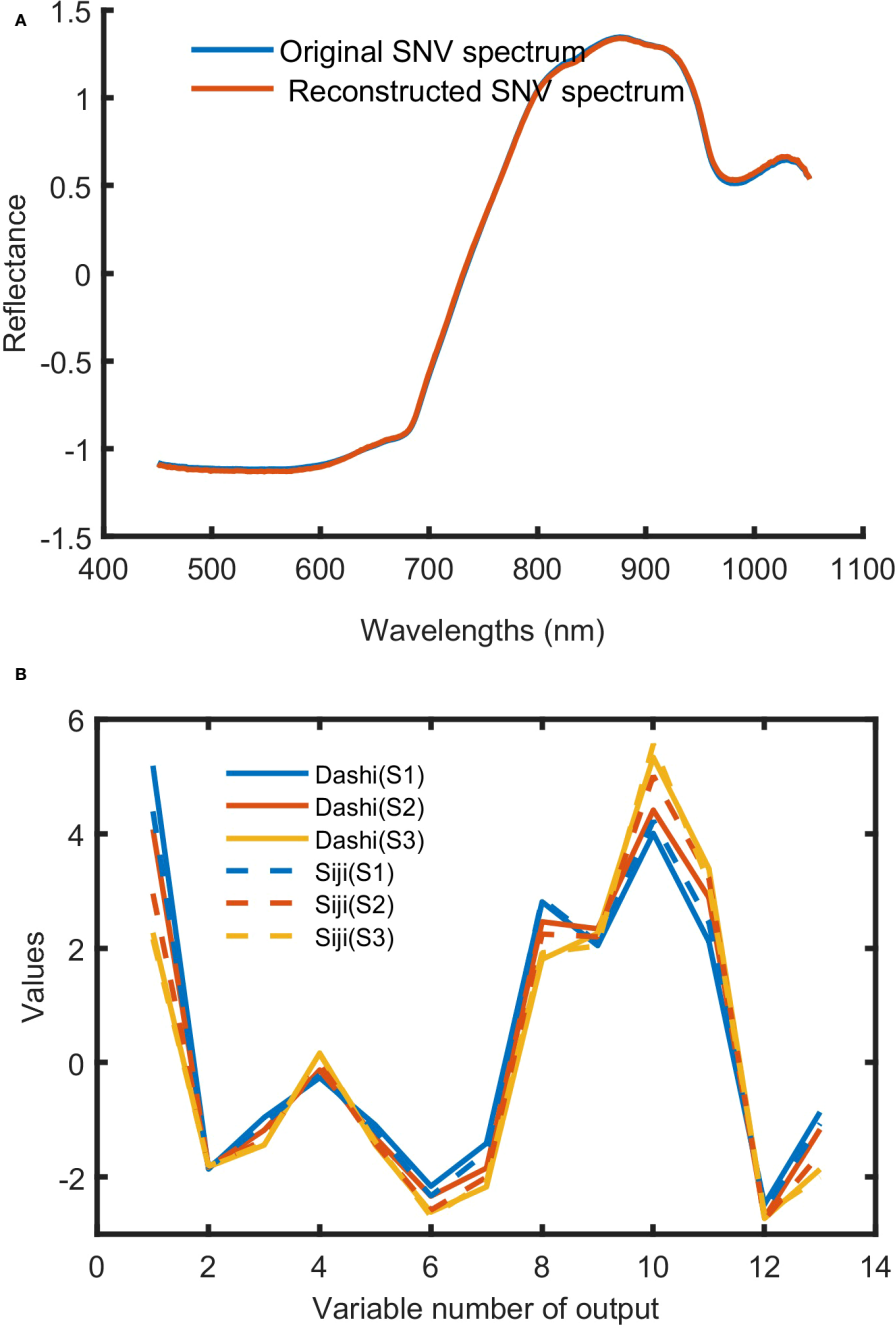
The training results of SAE. **(A)** Original SNV spectrum and reconstructed SNV spectrum; **(B)** deep spectral features of Dashi and Siji.

The feature variables of SAE screening are shown in **Figure 7**. When it comes to SAE, it is not necessarily the case that the more hidden layers are, the better the effect is. In this study, 379-300-150-h-150-300-379 was set to be the basic network. h denotes the number of neurons in the last coding layer, and it is also the number of feature variables extracted. Based on experience and many previous attempts, sigmod was set as the activation function, iterate was set to 40 times, the batch size was set to 200, the initial learning rate was set to 0.001, and h was set to 13. From the results shown in **Figure 7A**, the reconstructed spectral reflectance curve is highly coincident with the original spectral curve, indicating that the original spectral data can be perfectly reconstructed by using SAE. The last coding layer was extracted as the spectral feature variables (**Figure 7B**). It can be seen that the corresponding values of the 13 feature variables of samples at different maturity stages are obviously different.

### 3.3 The results of modeling

**Figure 8:**
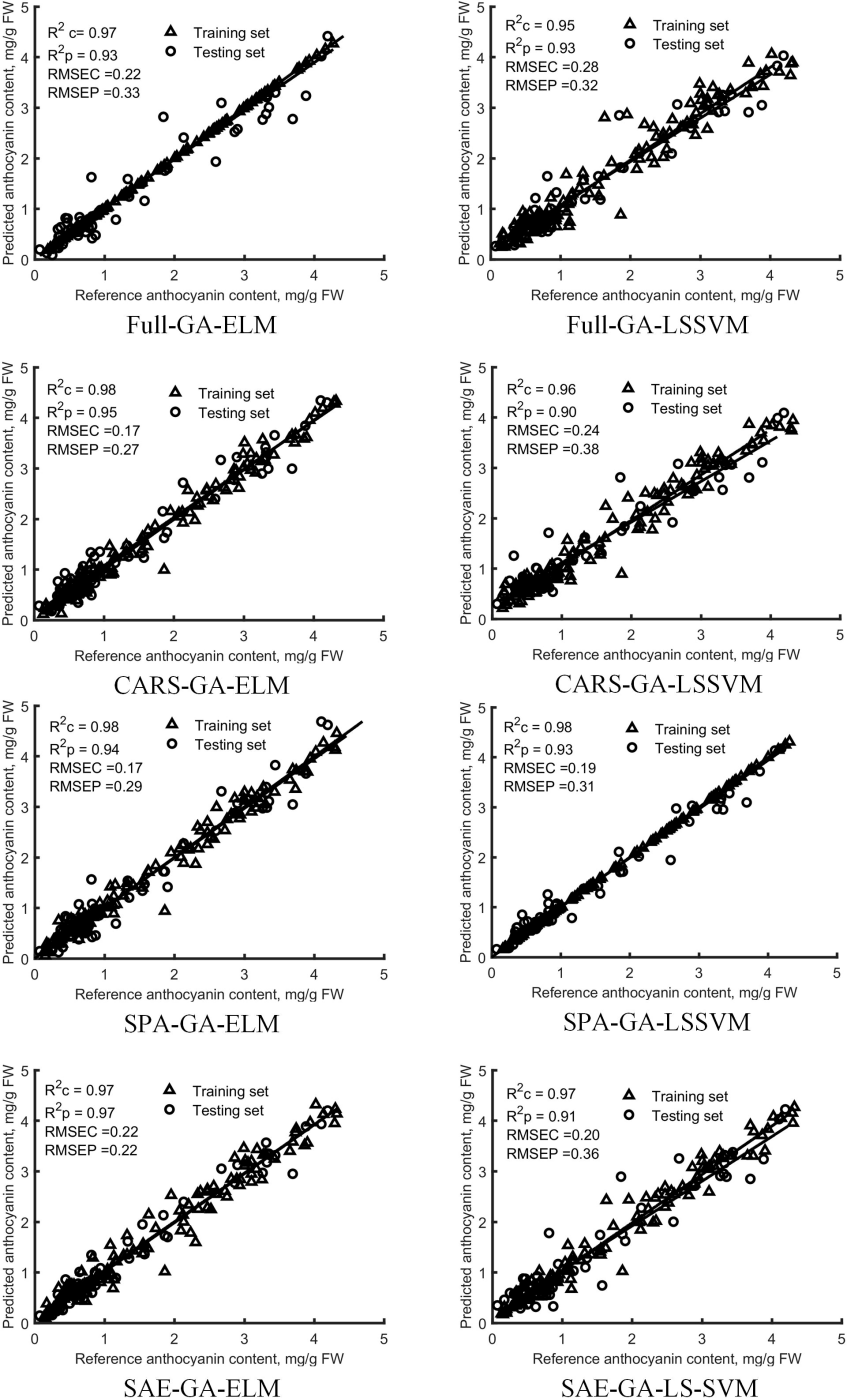
Diagnosis results of anthocyanin content in the training set and testing set by GA-ELM and GA-LS-SVM models based on all-band and feature variables.

All wavelengths and feature variables extracted by using SPA, CARS and SAE were used as the model inputs. Regression models of mulberry anthocyanin contents were built based on GA-LS-SVM and GA-ELM respectively. And the regression results were evaluated (**Figure 8**). Models were constructed by using the two non-linear regression methods that achieved good performance, R^2^ values of the training sets and those of the testing sets of GA-LS-SVM and GA-ELM models built on the basis of full wavelengths and variables extracted by using SPA, CARS and SAE were greater than 0.90, RMSE was less than 0.38 mg/g. The models based on variables selected by SPA, CARS and SAE achieved better performances than those based on full-band spectral data, indicating that SPA, CARS and SAE can reduce the redundancy of model input variables and help improve the accuracy of the model. Many researches show that ELM has the advantages of fast learning speed and good generalization ability (Wong et al., 2013; Huang et al., 2014). In this study, The SAE-GA-ELM models, requiring only 13 input variables, has achieved the best predictive performance, with the values of R^2^c and R^2^p reaching 0.97, and RMSEC and RMSEP being only 0.22 mg/g, obtained.

### 3.4 Visualization of anthocyanin content

**Figure 9:**
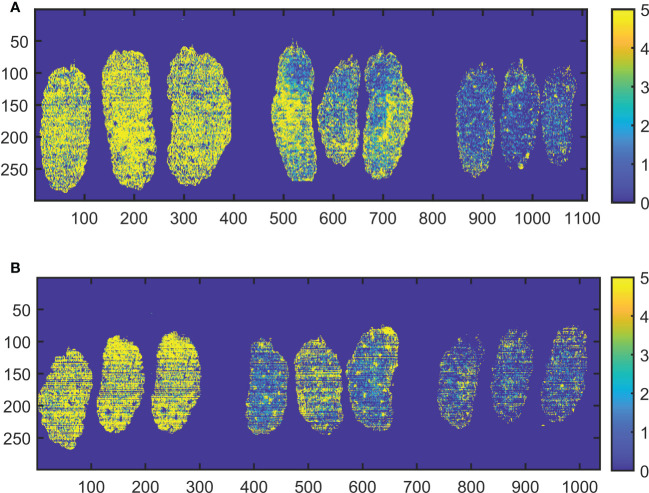
Visualization of anthocyanin content in mulberry fruits of Dashi **(A)** and Siji **(B)** at three maturity stages.

The visualization of anthocyanin content distribution in mulberry fruits is needed for more intuitively observing the changes in anthocyanin contents of mulberry fruits at different maturity levels. One of the advantages of hyperspectral imaging is that spectral data of each pixel can be obtained by using hyperspectral imaging. This makes it possible for the prediction about each pixel to be made, thus helping create distribution prediction maps. The visualization can be achieved with the average spectra applied for modeling and all of the single-pixel spectra in the hyperspectral image used for the best prediction model (Sun et al., 2019; Xiao et al., 2020). In this study, SAE-GA-ELM, the best model for anthocyanin content detection, was applied to visualize anthocyanin content distribution. All the single-pixel spectrum was processed by the same treatment used in the modeling. **Figure 9** shows the visualization maps of eighteen samples representing different maturity levels of two varieties. we can see from **Figure 9** that the higher the maturity level of mulberry fruits is, the higher the anthocyanin content is, and that the anthocyanin content of Dashi is higher than that of Siji at the same maturity stage, which is consistent with the results shown in **Figure 4A**. It can be seen that the distribution of anthocyanin content of the mulberry fruits at the red maturity stage is not consistent with that of the content of the mulberry fruits at the red to purple maturity stage, which is speculated to be the result of the uneven distribution of such anthocyanin synthesis regulators as sugars and hormones in the fruits (Aramwit et al., 2010; Mo et al., 2022).

## 4 Conclusions

In this study, with Dashi and Siji mulberry varieties selected as research objects, and SPA, CARS and deep learning methods SAE used to screen out feature variables, models for predicting anthocyanin content in mulberry fruits are built based on GA-LS-SVM and GA-ELM. The SAE-GA-ELM has achieved the best performance with R^2^c and R^2^p reaching the value of 0.97 under the condition of RMSEC and RMSEP being only 0.22 mg/g. By applying this best model to each pixel of the mulberry fruit images, distribution maps are created for visualizing the changes in anthocyanin content of mulberry fruits at three maturity stages. The results indicate that the hyperspectral imaging, in combination with SAE-GA-ELM could realize the fast, non-destructive, and high-precision detection of anthocyanin content of mulberry fruits, which means a new reference for rapid and nondestructive evaluation of physiological traits for the breeding, cultivation, harvesting and selling of the fruits.

## Data availability statement

The raw data supporting the conclusions of this article will be made available by the authors, without undue reservation.

## Author contributions

XL completed data collection, model construction, and paper writing. ZW and FP helped to collect data and provided comments and suggestions to improve the manuscript. JL edited the manuscript. GH directed the paper revision and provided the main idea. All authors contributed to the article and approved the submitted version.
